# Elective affinity or comprehensive contradiction? Reflections on capitalism and democracy in the time of finance-dominated accumulation and austerity states

**DOI:** 10.1007/s11609-018-0371-9

**Published:** 2018-10-09

**Authors:** Bob Jessop

**Affiliations:** Department of Sociology, Bowland College, Lancaster University, LA1 4YN Lancaster, Vereinigtes Königreich

**Keywords:** Austerity state, Authoritarian statism, Exceptional state, Financialization, Liberal democracy, Neoliberalism, Political capitalism, Austeritätsregime, Autoritärer Etatismus, Ausnahmestaat, Finanzialisierung, Liberale Demokratie, Neoliberalismus, Politischer Kapitalismus, régime d’austérité, étatisme autoritaire, état exceptionnel, financiarisation, démocratie libérale, néolibéralisme, capitalisme politique

## Abstract

The often-asserted relation of formal adequacy or elective affinity between capitalism and democracy is historically contingent on both sides of the relation. First, it holds for what Weber called “formally rational capitalism” – which is the form that Marx had previously investigated in *Das Kapital* – rather than others, such as traditional commercial capitalism or politically oriented capitalism. Second, it holds only to the extent that “the comprehensive contradiction” identified by Marx at the heart of the democratic constitution can be resolved: the contradiction between a universal franchise that potentially gives subaltern classes control over legislative and executive powers and a constitution that protects property rights favourable to capital. Building upon Poulantzas, it is then argued that these conditions are being undermined by the rise of new forms of political capitalism, especially finance-dominated accumulation, that are facilitated in turn by the consolidation of both neoliberalism and “authoritarian statism”. This involves the intensification of “exceptional” elements in a formally democratic shell, and the emergence of a permanent state of austerity. The article concludes with comments on the limits of finance-dominated accumulation and the austerity state.

## Zusammenfassung

Die häufig unterstellte Wahlverwandtschaft bzw. formale Entsprechung von Kapitalismus und Demokratie ist beiderseits historisch variabel. Zum einen unterscheidet sie sich je nach Form des Kapitalismus: Auf das, was Weber unter „rationalem Kapitalismus“ versteht (was dem von Marx im *Kapital* untersuchten entspricht), trifft sie eher zu als auf „politischen“ oder „traditionellen Handelskapitalismus“. Zum anderen stimmt jedoch auch das nur, insofern der „umfassende Widerspruch“, den Marx im Zentrum der demokratischen Regierungsform ausmachte, bewältigt wird: jener Widerspruch zwischen universellem Wahlrecht, das den subalternen Klassen potentiell die Kontrolle von Legislative und Exekutive erlaubt, und dem verfassungsmäßigen Schutz von Eigentumsrechten im Interesse des Kapitals. Wie sodann im Anschluss an Überlegungen Poulantzas’ argumentiert wird, werden die Voraussetzungen für diese Bewältigung durch den Aufstieg neuer Formen des „politischen Kapitalismus“ untergraben, und hier insbesondere durch die Dominanz finanzgetriebener Akkumulation, die wiederum durch die Konsolidierung sowohl des Neoliberalismus wie auch eines „autoritären Etatismus“ befördert wird. Dies impliziert die Ausbreitung von „Ausnahmeelementen“ im formaldemokratischen Rahmen ebenso wie die Herausbildung eines permanenten Austeritätsregimes. Der Artikel schließt mit Überlegungen zu den Grenzen von finanzgetriebener Akkumulation und Austeritätsregimen.

## Résumé

L’affinité élective ou correspondance formelle fréquemment alléguée entre capitalisme et démocratie est contingente dans un sens comme dans l’autre. Cette relation dépend d’une part de la forme de capitalisme considérée ; elle se vérifie plutôt pour ce que Weber appelait le « capitalisme rationnel » (la forme de capitalisme qu’avait étudiée Marx dans *Le Capital*) que pour le « capitalisme marchand traditionnel » ou le « capitalisme politique ». D’autre part, cette relation ne se vérifie que dans la mesure où la « contradiction fondamentale » que Marx identifiait au cœur du gouvernement démocratique peut être résolue : la contradiction entre le suffrage universel, qui permet en principe aux classes subalternes de contrôler le pouvoir législatif et exécutif, et la protection constitutionnelle des droits de propriété favorable au capital. Cet article affirme dans le prolongement de réflexions de Poulantzas que les conditions de cette résolution sont sapées par la montée de nouvelles formes de « capitalisme politique », et notamment par la prédominance de l’accumulation d’origine financière, elle même favorisée par la consolidation du néolibéralisme et d’un « étatisme autoritaire ». Cette évolution se manifeste par l’extension d’ « éléments exceptionnels » dans un cadre formellement démocratique ainsi que par l’émergence d’un régime d’austérité permanente. Cet article se conclut par des réflexions sur les limites de l’accumulation d’origine financière et des régimes d’austérité.

## On Capitalism and Democracy

Advocates and critics of capitalism alike have long suggested that the liberal democratic republic “is the best possible political shell for capitalism” (Lenin 1964, p. 398). More precisely, they seem to be claiming that liberal democracy is the *formally adequate* form of state in social formations in which rationally organized, profit-oriented, market-mediated accumulation provides not only the dominant principle of economic organization, but also of societal organization (“Vergesellschaftungsprinzip”). It is formally adequate because, when supported by substantive democratic institutions, liberal democracy disguises class power more effectively than when the state apparatus is openly controlled by the dominant classes (or class fractions) and/or is administered by state managers who use state power for self-enrichment.

Arguments for the formal adequacy and elective affinity of capitalism and liberal democracy tend to highlight and idealize specific accounts of capitalism and the state. On the one hand, the former is conventionally defined as a system of production based on, first, private ownership and control of the means of production and, second, wage labour. Restated in Weberian terms, it involves the formally rational organization of production based on the generalization of the commodity form to labour-power together with trade relations that are oriented to free markets (Weber 1968, pp. 161 ff., 2003, pp. 272 f., 292 ff., 373 f.). Weber paid special attention to the conditions for the maximum formal rationality of capitalist accounting and also noted some of the ways in which it might be considered substantively irrational in economic and social terms. He also identified forms of “politically oriented capitalism” (Weber 1968, pp. 161 ff.) that depend on various forms of extra-economic coercion and would seem to be less compatible with liberal democracy. I assess these issues below.

In turn, liberal democracy is conventionally equated with a national territorial state based on the rule of law and universal franchise. Restated in Schumpeterian terms, liberal democracy involves intermittent, quasi-plebiscitary competition between circulating political elites for the votes of individual citizens (Schumpeter 2005, pp. 427 ff.; Weber 1994, pp. 220 ff.). Defined in this minimal, elitist manner, democracy has survived or emerged in most post-war advanced capitalist states. However, if we include the institutional and socio-cultural prerequisites of effective democratic accountability, the situation looks less positive – for liberal democracy has specific legal preconditions. These include institutionalized political freedoms (e.g., freedom of association, freedom of speech, free elections), a competitive party system, the at least potential circulation into and out of government office of “natural” governing parties (either alone or in coalition), parliamentary or equivalent control over the executive and state administration, and the responsiveness of legislators and the executive to the electorate and public opinion within the limits of the rule of law. Popular-democratic struggles aim to extend the sphere of validity of citizens’ rights, to include more of the population in the category of citizens, and to institute the legal framework for creating and maintaining the social conditions and balance of forces that enable the people to monitor and safeguard these preconditions.

The mutual relation between capitalism and democracy has so far been studied and rendered plausible mostly in terms of a synchronic formal isomorphism. Defenders of this claim have shown less interest in the historical trajectories of economic and political institutions and practices. Yet these trajectories show the contingency of that relation between capitalism and democracy and, where they co-exist, reveal its manifold structural contradictions, strategic dilemmas, and discursive paradoxes. These contingencies are summarized neatly in Stanley Moore’s aphorism that, “when exploitation takes the form of exchange, dictatorship *tends to take* the form of democracy” (1957, p. 85, ital. BJ). In short, the relation is not guaranteed.

Hence, even though the claim of an “elective affinity” between capitalism and democracy may sound plausible in abstract terms (Paschukanis 2003; Fine 1984) it ignores the deeply contradictory and historically contingent nature of the relation between economic and political institutions in capitalist formations. Indeed, recent trends are once again making capitalist domination more visible due to the rise of finance-dominated accumulation and other forms of political capitalism. In making this claim, I distinguish finance-led from finance-dominated accumulation. Whereas the former is based on the increased importance of capitalist credit relations in the circuits of capital, the latter involves the dominance of interest-bearing capital within the wider social formation as well as in the circuits of capital. This is not the result of spontaneous market forces but of a series of political interventions and of the resort to extra-economic power. Such developments are intensifying recent trends to authoritarian populism, authoritarian statism, and “post-democracy”. Moreover, in response, we observe various forms of popular reaction ranging from right-wing extremism through political apathy to radical left-wing populism or ecosocialism.

Therefore, it needs further consideration whether the relation between capitalism and democracy is facing new challenges in the era of finance-dominated accumulation regimes operating in the shadow of neoliberalism – and, if so, what this implies for the form of state and its role in securing economic exploitation and political domination. Based on the definitions set out in this section, the rest of this article critically interrogates the claim that there is a formal correspondence or elective affinity between capitalism and democracy. Section two qualifies and expands on the definitions, noting the significance of political capitalism in capital accumulation, and then explores the historically contingent conditions in which this “fit” emerges and might prove stable. Section three extends this analysis by building on Marx’s analysis of the “comprehensive contradiction” in the bourgeois democratic constitution and by outlining the significance of the institutional separation between the economic and political spheres as an important condition of relatively stable accumulation and bourgeois democracy – in large part because it constrains the forms of economic and political struggle to the advantage of capital. Section four suggests that, in the absence of these conditions, various political crises emerge and, depending on the balance of forces, may lead to attempts to weaken or suspend the electoral principle that is crucial to liberal democracy and establish one or another form of “exceptional regime”. Assuming this, section five introduces Poulantzas’s argument that “authoritarian statism”, which is characterized by the intensification of “exceptional elements” within a formally democratic political regime, has become the “new normal” form of state. Building on and updating this argument, section six posits that it is mainly the increasing importance of finance-dominated accumulation – facilitated by neoliberalization – that both undermines formally rational capitalism and intensifies the trend towards authoritarian statism. Section seven indicates how this transforms the comprehensive contradiction in the democratic constitution compared to the mid-19^th^ century when Marx identified it. For the conflict between political restoration and social emancipation is being resolved in favour of capital rather than in favour of the subaltern classes. Section eight illustrates this by following the shift from austerity policies through neoliberal politics of austerity up to the consolidation of a polity of permanent austerity. Finally, section nine provides some brief reflections on the prospects for social emancipation.

## “The best possible political shell”?

The claim that democracy is the best possible political shell of capitalism depends on a formal isomorphism between formally rational capitalism and the form of the liberal democratic state. But it is clearly questionable on at least two grounds. First, formally rational capitalism is not the only basis of capital accumulation and other bases may be less compatible with liberal democracy. This is clearly the case with “primitive accumulation” and imperialism and, as we shall see, also holds for more recent forms of political capitalism. Second, authoritarian rule that represses or otherwise restricts popular economic and political mobilization has been a crucial factor in capital accumulation. The literature on developmental states provides many examples. Political theorists note the potential disjunction between capitalism and democracy when they distinguish “normal” – that is, democratic – capitalist states from “exceptional” political regimes that abolish free elections in favour of executive authority (Poulantzas 1974). This indicates a need to explore the historical articulation of capitalist development and the modern state and the variability of capitalism and state forms.

Regarding capitalism, the formally rational organization of production and continuous buying and selling on the market with free exchange that corresponds formally to liberal democracy comprises only one of two modes of formally rational orientation to profit (“Erwerb”) that Max Weber identified from his historical studies of capitalist development (Weber 1968, pp. 164 f.). Note here that formal rationality involves numerical – here: monetary – calculation about potential and actual profit-and-loss (Ibid., pp. 85 f.) and has no implications for the substantive rationality of capitalism, including economic, social, or, one might add, environmental justice. The second formally rational mode involves trade and speculation on organized exchanges in graded or otherwise standardized commodities, securities, futures, initial public offerings of listed companies, and the financing of political bodies (Weber 1968, pp. 164 f.; 2003, pp. 250, 295, 303, 311). Another mode identified by Weber is traditional commercial capitalism based on trade and speculation in currencies, financial intermediation, and usury for production, trade, or consumption purposes, as well as the buying and selling of commodities. While some of these activities might seem to overlap with the preceding kind of orientation to profit, they do not benefit from the conditions for maximum formal rationality (of calculation of opportunities for profit) that exist in modern rational capitalism (Weber 2003, p. 370).^1^

Weber also identified three ideal-typical, albeit internally heterogeneous, modes of “politically oriented capitalism” based on different forms of extra-economic coercion (Weber 1968, p. 166). These comprise, first, an orientation to predatory profit from political activities, including the financing of wars, revolutions, or party leaders; second, profits from continuous business activity based on force or a monopoly granted by political authority in the form of colonial property or fiscal revenues from tax farming or sale of offices; and, third, an “orientation to profit opportunities in unusual transactions with political bodies” (Weber 1968, pp. 164 f.; see also Weber 2003, pp. 75 f., 94 ff., 278, 281 f., 296, 300, 315 f., 353 f., 370; Swedberg 1998, pp. 48 ff.).^2^ Weber’s analysis has an historical dimension. For, whereas the different forms of political capitalism and traditional commercial capitalism have long histories, formally rational capitalism can, according to Weber, only become dominant and shape the dynamic of capital accumulation when economic activities are disembedded from traditional social relations and when other spheres of society have undergone rationalization.

Consistent with this interpretation to be qualified below, several theorists have suggested the following periodization. First, the origins of capitalism in Western Europe were tied to mercantilism and absolutism and the top-down exercise of state power to promote primitive accumulation and establish the capital-labour relation. Second, with the consolidation of competitive capitalism in the first wave of capitalist economies, a liberal state secures the general conditions for accumulation to facilitate free market competition and intervenes when necessary to redress the effects of unfettered competition (e.g., factory legislation). This state is typically based on the rule of law and parliamentary representation of propertied interests. Extending legal and political rights and the franchise reinforces the illusion of equality among citizens to match the illusion of equality between buyers and sellers of labour-power. But the commodity fetishism of free markets and the political fetishism of a constitutional state cannot ensure a stable fit between free markets and liberal democracy. As voters elect large numbers of social democratic and communist deputies, parliament is less able to arbitrate among competing capitalist interests and this task is therefore transferred to the executive. Third, as monopoly capitalism drives out liberal competitive capitalism and/or capital’s crisis-tendencies intensify, the state is pressured to intervene to renew accumulation, often at the cost of subaltern classes (cf. Holloway and Picciotto 1977; Mandel 1972; Gerstenberger 2011). Moreover, as Alexander Gerschenkron (1962) and others note, banks and the state had bigger roles in second-wave industrialization with the result that competitive capitalism was weaker, even where it existed, and political capitalism had a bigger role. This leads to a strong state with fewer opportunities for popular participation in policy formation.

This simplified three-stage model holds mainly for first-wave capitalist economies, such as England, the Netherlands, Belgium, and the USA. Even these cases also involved political capitalism (e.g., the financing of wars, imperial conquest, colonialism, the slave trade and slavery, state-sponsored monopolies, and forms of political corruption). In late development, the phases of mercantilism – with or without absolutism – and interventionism tend to be merged and liberal states are absent or ineffective. Examples include Bonapartism, Bismarckism and Tsarist autocracy. Similar points hold for East Asian developmental states and their successors in third-wave industrialization and for many post-socialist “transition states”, notably those that are resource-rich. As developmental states sought to catch-up with advanced economies through export-led growth, economic growth often relied on low-waged, labour-repressive primitive accumulation.

Several important accounts also suggest that “authoritarian” forms of rule are characteristic of mature capitalism and not confined to periods of primitive accumulation, late development, or dependent and peripheral capitalisms. Examples include the ideas of first generation Frankfurt School theorists on trends towards a strong, bureaucratic state – whether authoritarian or totalitarian in form – in the context of economic crisis (Dubiel and Söllner 1984; Scheuerman 1996; and the essays in Scheuerman 2008). Early Frankfurt School theorists argued that this state form was linked to the rise of organized or state capitalism, which relied increasingly on the mass media for its ideological power and which either integrated the labour movement as a political support or smashed it as part of the consolidation of totalitarian rule. The theme of authoritarian rule was revived again following the end of the Second World War, especially in the context of the Cold War. It was associated with the rise of the national security state and the crisis of the post-war Atlantic Fordist mode of growth, which had combined rising prosperity with strong support for catchall parties and an expanding welfare state.

This suggests that it would be mistaken to think that the political modes of securing profit belong to the past. Indeed, recognizing that they survive and may even be expanding, albeit in new as well as old forms, might lead one to different conclusions about the formal correspondence between capitalism and democracy. Indeed, one might well propose that, where political forms of profit-making are dominant, it is authoritarian rule that is the norm rather than the exception (Jessop 2013, 2015c).

## The comprehensive contradiction in bourgeois democracy

This brief discussion of political capitalism and the accompanying historical summary indicate that the adequacy of the bourgeois democratic republic depends on the overall economic, political and ideological situation and its implications for the equilibrium of compromise necessary for the democratic constitution. Thus, commenting on the French constitution in 1850, Karl Marx noted a “comprehensive contradiction”: “The classes whose social slavery the constitution is to perpetuate, proletariat, peasantry, petty bourgeoisie, it puts in possession of *political power* through* universal suffrage*. And from the class whose old *social power* it sanctions, the bourgeoisie, it withdraws the *political guarantees* of this power. […] From the ones it demands that they should not go forward *from political to social emancipation*; from the others that they should not go back *from social to political restoration*.” (Marx 1978, p. 79, ital. BJ)

This argument can be related to a common theme in analyses of the liberal democratic republic: namely, that the separation of the economic from the political sphere limits the concentration of power to the mutual benefit of economic and political agents. Economic power can be used to prevent the abuse of political power, whereas political power can be used to counteract market failures. According to bourgeois apologists, where this separation exists competitive capitalism can expand through the smooth operation of market forces, whilst, in addition, dispersed economic power helps to block the abuse of political power. Milton Friedman (2004), a neoliberal ideologue, even claimed that an authoritarian capitalism was preferable to totalitarian communism because, at least, capitalism sustained economic freedom. Radical political economists and political theorists dispute this, however, arguing that competitive capitalism and liberal democracy are the twin supports of capitalist rule (Macpherson 1973, pp. 148 f.). Both views can be related to Marx’s analysis of democracy.

The comprehensive contradiction that he identified suggests that one crucial material basis for the reproduction of liberal democracy is the institutional separation between the economic and political in capitalist societies and its reflection in a clear demarcation between economic and political class struggle. For capital in general, the ideal position is one where class struggle is confined within the limits of the market relation and political class struggle occurs within those of bourgeois parliamentarism. Thus, subaltern classes should focus economically on improving or defending wages and conditions, politically on mobilizing public opinion and securing parliamentary majorities to advance or defend social reforms. Trade unions should not engage in strikes, especially general strikes, to support parliamentary or wider political action. Subaltern classes can participate in the political process on condition that they do not use their political – read electoral and parliamentary – power to challenge the social – read economic, political, and ideological – power of the dominant classes. Subaltern political action should be limited to influencing the “public” sphere and refrain from directly constraining the rights of private property, especially the allocation of capital to alternative investments and management of private enterprise. Where this kind of institutional separation is reproduced, subaltern classes find it hard to mobilize their full potential for defensive and offensive collective action. Moreover, where capitalism rests on equal exchange, trade in free markets and rationally-organized production, and the state’s revenues and credit-worthiness depend on taxes levied on private economic agents, the bourgeoisie has no need to control the state directly provided that the latter maintains the juridical, monetary, and other extra-economic conditions for accumulation and capital can engage in investment strikes or move capital abroad.

Liberal democracy is significant here because it facilitates the *disorganization *of subaltern groups. Because individual citizenship is based on the formal equality of all adult members of society, their common interests are expected to cut across class antagonisms (Poulantzas 1973, 1978). Civil society is fragmented, even individuated, and the legally sovereign state is expected and empowered to define and promote the “national interest” and the “public good”. Further, where surplus-labour is appropriated through market forces rather than coercion, capital can offer political concessions, such as welfare state benefits, without threatening accumulation. The rise and expansion of the welfare state reinforces the potential mutual isolation of citizens as clients or consumers of distinct, multi-scalar public services. “The people” is thereby divided into heterogeneous client groups competing for state resources.

Conversely, the dominant classes can enjoy their social power as long as they tolerate the short-term vagaries of democratic rule. Liberal democracy is advantageous here because it facilitates the organization of the power bloc: different fractions have the chance to bargain, compromise, and adjust their interests in ways that promote their long-term interests and, if possible, offer concessions to subaltern groups. As Antonio Gramsci (2012) noted, this leads to struggles for hegemony (in the sense of political, intellectual, and moral leadership) as dominant classes and their political allies develop economic strategies, state projects and hegemonic visions that reconcile, partially, the diverse interests of different economic, social, and other categories in a supposed general interest. “Natural governing parties” have a key role here whenever they can reconcile the interests of a substantial part of the electorate and key sections or fractions of the dominant classes (Gamble 1973).

In these conditions, universal suffrage, competing parties, the separation of powers, and parliamentary government all contribute to the flexibility of a political system, meaning that power can be continually readjusted to secure social cohesion. And since the cohesion thereby secured is that of a class-divided society, this also maintains class domination. Indeed, workers’ struggles can sometimes impose policies that advance the long-term interests of capital. Two examples are 19^th^ century factory legislation (Marx 1972, pp. 505 ff.) and the Cold War class compromise that facilitated a virtuous circle of mass production and mass consumption in the Atlantic Fordist boom (Poulantzas 1973, 1975).

This point can be linked to the administration of the modern state. The latter involves a formal separation between partisan politics oriented to struggles to define the – always illusory – national-popular will, and non-partisan administration that impartially implements political decisions. Whether or not this formal separation is effective is often hidden behind a cloak of official secrecy, intransparency, and control over information flows on a “need to know”-basis. In addition, bureaucratic domination separates citizens from direct control over the means of administration. This holds as much for economic intervention and welfare administration as for the means of coercion and repression and reproduces citizens’ dependency on efficient administration.

The flexibility inscribed in the normal democratic state, especially through the turnover of political parties and coalitions, often provides the basis for crisis-management or, at least, the ability for a “fuite en avant”, i.e., a continual game of blame, displacement, and renewed disappointment. However, there is no guarantee that the “comprehensive contradiction” can be managed in these or other ways. Political crises may occur where the institutional separation between economic and political struggles necessary to the smooth and legitimate functioning of liberal democracies breaks down – for example through a general strike with political objectives and/or the use of political power to expropriate capital or challenge its prerogatives. Likewise, they may emerge if the link between the economic and political spheres breaks down because economic or fisco-financial crises limit the scope for material concessions to subaltern groups, leading to greater reliance on passive revolution, corruption or coercion to secure political authority.

## Political Crisis, States of Emergency and Exceptional Regimes

During periods of political crisis, the “comprehensive contradiction” of liberal democracy prompts open struggles between subaltern and dominant classes over the nature and ends of government. Notions such as Bonapartism and Caesarism were integral parts of 19^th^ century European political discourse and provided a focus, alongside that of democracy, for exploring the relation between political authority and the popular will. This theme survived into the 20^th^ century, especially during the interwar period around dictatorship and totalitarianism. On this basis some scholars, orthodox and heterodox alike, distinguish normal states and exceptional regimes in terms of conformity to democratic institutions and hegemonic class leadership. Normal states characterize conjunctures in which bourgeois hegemony is stable and secure, whereas exceptional regimes develop in response to crises of hegemony. Thus, while consent predominates over constitutionalized violence in normal states, exceptional states intensify physical repression and conduct an “open war” against dominated classes, moving towards a police-military resolution (Gramsci 1971, pp. 210 ff., 179 ff.).^3^

Relying on Gramsci’s analysis, a substantial crisis within the political sphere can arise for a number of reasons: when the representational ties between major parties and their supporters are broken; when there is a crisis within the power bloc; when the institutional integration of the state dissolves, leading to incoherent or contradictory policies; when state intervention becomes ineffective, leading to a rationality crisis; when there is a loss of political legitimacy; when a crisis of hegemony emerges; or when, based on interaction among some or all of these crises, an organic crisis of society eventually develops. Vice versa, three common responses to such crises of the state are: first, to reorganize the system of representation, especially its electoral aspects, to weaken the prospects of radical, popular-democratic or socialist governments. Second, to promote governments of national unity based on cooperation among the natural governing parties and the co-option or suspension of other parties. And, third, to limit the powers of parliament and elected officials by reinforcing the independence of key administrative apparatuses (e.g., central banks, the security apparatus) and/or declaring temporary states of economic or political emergency. Where such responses cannot be implemented or subsequently fail, attempts are often made to suspend or eliminate democratic institutions. This can lead to an exceptional regime that reorganizes state structures and the balance of forces – or else end “in the common ruin of the contending classes” (Marx and Engels 1976, p. 482).

According to Poulantzas, though, only one type of political crisis produces an exceptional political regime: namely, a crisis of hegemony within the power bloc. This occurs when no class or fraction can impose its “leadership” on other members of the power bloc, whether by its own political organizations or through the “parliamentary democratic” state. This is typically related to a general crisis of hegemony over the whole society. Such crises are reflected in the political scene and the state system. Symptoms include: a crisis of representation, attempts by various social forces to by-pass political parties and influence the state directly, and efforts by different state apparatuses to impose political order independently of decisions coming through formal channels of power. Such phenomena can undermine the institutional and class unity of the state even where it continues to function and provoke splits between top echelons in the state system and lower ranks. The state may also lose its monopoly of violence (see Poulantzas 1974, passim; 1976, p. 28).

Faced with such political crises, the democratic rule typical of the normal state may be suspended and states of emergency or exceptional regimes introduced. This distinction between normal state and exceptional regime derives from the legal justification of states of emergency as being limited in duration until the crisis was overcome. For Poulantzas, it also reflected the European experience concerning the instability of most exceptional regimes. In this context, he identified five major contrasts between normal states and exceptional regimes:

**Table 1:** Normal States and Exceptional Regimes (Source: based on Poulantzas 1974, 1976, 1978)

Normal States	Exceptional Regimes
Liberal democracy with universal suffrage and formally free elections	Elections are suspended (except for plebiscites and referenda)
Power is transferred between parties and/or governments in a stable way in line with rule of law	No legal regulation of power transfer (“might is right”, state of exception, state of siege)
Pluralistic series of ideological apparatuses that operate relatively independently of the state	Ideological apparatuses are integrated into the official state to legitimate its enhanced power
Separation of powers	Concentration of powers
Power circulates organically, which facilitates a flexible reorganization of power	These regimes congeal balance of forces existing at the time that an exceptional regime is introduced

Poulantzas’s analysis of how exceptional regimes congeal the balance of forces that prevailed when they are established echoes an earlier remark by Hannah Arendt. She noted that, once they have seized power, dictatorships tend to become routinized, predictable, and domesticated (1956, p. 407). The freezing of a given conjuncture makes it harder to resolve new crises and contradictions through routine and gradual policy adjustments and to secure a new equilibrium of compromise (Poulantzas 1974, 1976). In short, the alleged strength of an exceptional regime hides brittleness. Yet Poulantzas discerned important differences among exceptional regimes and was particularly impressed by the flexibility and manoeuvrability of fascism. In contrast, military dictatorship is the least flexible type and Bonapartism is located halfway between these extremes (for further discussion, see Jessop 1985, pp. 229 ff.). Arendt drew a similar distinction between dictatorships, which tended to stagnate, and totalitarian states, which, she claimed, were in a constant state movement, transgressing barriers, and engaged in permanent revolution (cf. Canovan 2004).

The relative rigidity of exceptional regimes is especially true, Poulantzas argued, where they lack specialized politico-ideological apparatuses to channel and control mass support and are thereby isolated from the masses. He illustrated this in terms of the contrast between the fascist regimes in Italy and Germany and military dictatorships. The latter are marked by a rigid apportionment of state power among distinct political clans linked to each apparatus. They have no ideology that can forge the necessary state unity and can establish an effective national-popular cohesion. This produces a muddle of inconsistent policies toward the masses as the exceptional regime attempts to neutralize its opposition. It also leads to purely mechanical compromises, tactical alliances, and settling of accounts of their “economic-corporate” interests among the dominant classes and fractions. In turn this intensifies the internal contradictions of the state apparatus and reduces its flexibility in the face of economic and/or political crises (1976, pp. 83 f.).

This analysis was inspired by the collapse of the dictatorships in Greece, Portugal and Spain in the mid-1970s. The collapse of the socialist states in Central and Eastern Europe in the 1980s provides other examples. Thus, just as the movement from a normal to an exceptional state involves political crises and ruptures rather than taking a continuous, linear path, so the transition from an exceptional to a normal form will also involve a series of breaks and crises rather than a simple process of self-transformation. This places a premium on the political class struggle to achieve hegemony over the democratization process.

## From parliamentary democracy to authoritarian statism

In the 1970s, Poulantzas described the “new normal” form of capitalist state as involving the “irresistible rise” of authoritarian statism. He argued that the capitalist type of state is now “*permanently and structurally characterized by a peculiar sharpening of the generic elements of political crisis and state crisis*” (1978, p. 206). This reflected the long-term structural economic crisis of contemporary capitalism that was manifest in the 1970s and its reflection in a variety of political and ideological crises that were fracturing the social bases of the interventionist state, including the decomposition of the traditional alliance between the bourgeoisie and the old and new petty bourgeoisie, the growing militancy of rank-and-file trade unionists and other subaltern groups, the ideological crisis accompanying the growth of new social movements on erstwhile “secondary” fronts, and the sharpening of the contradictions within the power bloc because of the impact of internationalization on the relations between fractions of capital (1978, pp. 210 ff., 219, 221). These symptoms reflect the crisis of Atlantic Fordism. But analogous symptoms can be seen in the export-oriented knowledge-based economies and neoliberal finance of the 1990s and later. Thus, although Poulantzas’s account is marked by the conjuncture of the 1970s, it can be reworked for the rise of neoliberalism in a far more integrated world market and its manifold crises in the current period.

For Poulantzas, continuing the earlier metaphor, authoritarian statism had become “the best possible political shell for capital”. Political features that were previously exceptional and temporary were being normalized in this type of capitalist state. Its basic tendency is “intensified state control over every sphere of socio-economic life combined with radical decline of the institutions of political democracy and with draconian and multiform curtailment of so-called ‘formal liberties’” (1978, pp. 203 f.). He related this mainly to the state’s increasing assumption of economic functions as modified by the political situation to tame the “wilder” manifestations of capital’s crisis-tendencies – witness the Great Depression –, promote international competitiveness, and extend the profit-oriented, market-mediated logic of capital accumulation into ever more spheres of social life (1978, pp. 163 ff.). For, as the world market has become more integrated, its contradictions have been generalized and its crisis-tendencies have become more evident. This makes it harder to displace or defer crises, so they become a generally visible permanent feature of contemporary capitalism. Thus, significant “exceptional” features are orchestrated into a permanent structure that runs parallel to the official state system and co-exists with and modifies the “normal” features of the capitalist state in a symbiotic, intersecting system managed by the natural governing party or parties and the power centres inside the state apparatus.

More specifically, drawing on several of Poulantzas’s analyses, “authoritarian statism” can be said to have six key features, with the exceptional features of authoritarian statism being articulated under the dominance of the normal elements. First, the crisis of hegemony means that the state administration becomes the *central *site at which the unstable equilibrium of compromise within the power bloc is elaborated. The legislative, executive, and judicial branches are increasingly fused, with real power being concentrated and centralized in the administration and sealed off from serious influence by parties and parliaments. Politics is now increasingly focused in and on the staff office of a president or prime minister. These leaders may appear to exercise despotic personal power, but they more often serve as a charismatic figurehead who gives a sense of strategic direction in a complex, crisis-prone world. Personalism condenses many contradictory pressures and works to re-balance conflicting forces. Popular interests nonetheless still surface in the form of contradictions inside the administration (1978, 1974, pp. 311 ff.).

Second, there is a decline in general, formal, and universal legal norms enacted by a parliament with a popular mandate that embodies the general will of the people-nation. Instead, legal norms are set by the political executive and administration in response to particular conjunctures, situations, and interests and in consultation with business interests and associations – especially regarding economic intervention, broadly interpreted (Poulantzas 1978, pp. 218 f.; Scheuerman 2003). Likewise, as monopoly capital finds it hard to organize its hegemony through parties other than the dominant mass party or set of natural governing parties, it also relies on an expanded lobby system to influence the administration. Recent research on the U.S. case shows that economic elites and organized groups representing business interests have substantial independent impacts on U.S. government policy, while ordinary citizens and mass-based interest groups have little or no independent influence (Gilens and Page 2014; see also Ferguson 1995; Hacker and Pierson 2011). Exemplary here is the role of business lobbies, such as the American Legislative Exchange Council (ALEX), which prepares boilerplate model legislation for rolling out at state level in the U.S. More generally, one might add that their elaboration is increasingly delegated to non-accountable private authorities at different scales up to and including the global (Cutler 2009; McKeen-Edwards and Porter 2013).

The decline of the rule of law also affects the political sphere. One sign of this is the increasing emphasis on pre-emptive policing of the potentially disloyal and deviant rather than the judicial punishment of clearly defined offences against the law (Poulantzas 1978, pp. 219–220; Boukalas 2014). This is reflected in the increasing importance of the (national) security apparatus, the way its operations cross-cut formal bounds and boundaries within the state, and how it is linked through parallel power networks to important forces formally located beyond the state. Some of its strategies and tactics were pioneered in colonies, the (semi-)periphery, or occupied countries (e.g. McCoy 2009; Grandin 2007). The threat of terrorism is invoked to roll out the state with the result that “[t]he only way that terrorism affects our daily lives is through counterterrorism” (Boukalas 2014, p. 2).

Third, the legislature becomes a mere electoral “registration chamber” with very limited powers and the state administration, guided by the political executive, becomes the main site for developing state policy. Concomitantly, fourth, the “natural parties of government” – in contrast to parties destined for a permanent oppositional role – no longer fulfil their traditional functions in policy-making by building compromises around a common programme or political legitimation through electoral competition for a national-popular mandate. Now their function is to transmit the state ideology to the popular masses, and to mobilize mass support for state policies in a plebiscitary fashion (Poulantzas 1978, pp. 236 f.; see also Blyth and Katz 2005). They engage in electoral manipulation based partly on cultivating close ties to the mass media, which promotes legitimation through a “populist ventriloquism” that speaks in the name of the “people” and thereby creates the public opinion to which politicians respond (Poulantzas 1978, p. 229; Hall et al., 1978, pp. 77 ff., 125, 325). Political parties thereby lose their role as the leading channels for political dialogue with the administration and as the major forces in organizing hegemony, and become transmission belts for official decisions and amplifiers of populist ventriloquism (Poulantzas 1978, pp. 229 f., 237). This can occur in two ways: through a long period without alternation among the governing parties or through the development of a single inter-party ‘centre’ that dominates the alternating parties of power.

Fifth, parties’ ties of representation to the power bloc become looser because monopoly capital finds it harder to organize its hegemony through parliamentary parties and concentrates its lobbying on the administration (1974, 1975, 1978, pp. 221 ff.). This further undermines the already limited involvement of the masses in political decision-making, severely weakens the organic functioning of the party system even where a plurality of parties survives intact, and saps the vitality of democratic forms of political discourse. This makes it easier for the state to penetrate into all areas of social life – especially, one might add, where this is justified in the name of (national) security and a war on terrorism.

Sixth, this massively politicizes the administration and risks its fragmentation behind a formal façade of bureaucratic hierarchy and unity (1978, p. 236). Accordingly, a “parallel power network” cross-cuts the formal divisions of the state and wields a decisive role behind the scenes on behalf of monopoly capital in coordinating official, semi-official, and “private” activities across various policy fields (Poulantzas 1974, 1978). With a strong anti-democratic ideology, these networks contribute to the evolving strategic line of the dominant mass party when it is in power and play an obstructive role if a radical party wins office (Poulantzas 1978, pp. 203 ff.; for similar arguments, see von Armin 2001; Greven 2010).^4^

While this summary may imply that Poulantzas believed that the “authoritarian statist” path runs smoothly, he stressed that state power continually runs up against limits inherent in its political matrix and operations, reflected in internal divisions and political resistance, as well as limits posed by the growing incompressibility of economic contradictions and crisis-tendencies and the challenges from new forms of popular struggle. Accordingly, he argued that this trend involves a paradoxical strengthening-weakening of the state. Muddling through, crises of crisis-management, and pre-emptive policing of resistance are symptoms of the incompressibility of capital’s contradictions and the intensifying crisis-tendencies of an increasingly integrated world market (Poulantzas 1978, pp. 241 ff. and passim). The state must either allow economic crises to run their course or assume responsibility for managing them and displacing or deferring their effects without eliminating them. It is also much harder for the dominant fraction to sacrifice its short-term economic-corporate interests to promote its long-term political hegemony – especially in the absence of a competitive party system and in the face of the growing internationalization of capital, which limits the state’s temporal and territorial sovereignty. This creates problems of national unity that are especially clear in less developed regions and/or its impact on national minorities (Poulantzas 1978, p. 213). Likewise, the state’s growing involvement in hitherto marginal areas of social life politicizes the popular masses and provokes new social movements. It is a state-form which results from the need of the state to combat economic and political crises – but it is also a form that generates crises and thereby creates the conditions for further extension of the security state.

## Finance-dominated accumulation

The rise of finance-dominated accumulation extends and deepens forms of political capitalism in advanced post-war societies and leads to major changes in the circuits of capital as interest-bearing capital forms closer ties to the state apparatus – for neither neoliberalism in general nor finance-dominated accumulation in particular is a spontaneous result of market forces. They are planned outcomes of political deal-making, corruption and “revolving doors”: an instance of relative fusion of economic and political power within the dominant classes. This, in turn, creates the conditions for accumulation based on force, predation and primitive accumulation and, when crises occur, for rescuing financial institutions that are too big, too systemically important, or just too well-connected to be allowed to fail, as well as for passing the costs to the “little people”. The resulting crisis of bourgeois political hegemony, whether based on claims to democratic legitimacy or on delivering growth and prosperity for all or most citizens, is nonetheless combined with a remarkable survival of bourgeois political as well as economic domination (Crouch 2011; Konings 2018).

This paradox is related to the further extension of a “post-democratic” authoritarian statism. Capitalism’s elective affinity with liberal democracy is weakened when profits derived from financial speculation and risk-taking start to exceed those that come from the financial intermediation and risk-management activities that are essential to the circuits of productive capital. It is further weakened where finance-dominated accumulation leads to growing inequalities in income and wealth due to deregulation, liberalization, and the interpenetration of economic – especially financial – and political power. And it is even less sustainable when the dominant forms of orientation to profit depend on predatory political profits (including kleptocracy and primitive accumulation based on dispossession), profit on the market from force and domination (including use of state power to impose neoliberal rules, institutions and practices on other accumulation regimes and open up new fields of accumulation), or “unusual deals with political authorities” (such as financial contributions for special legislative, administrative, judicial, fisco-financial or commercial decisions that privilege particular capitals and fall well outside the normal working of the rule of law). I leave aside here predatory capitalism associated with the conduct of “wars”, whether actual wars of conquest, plunder or colonization or metaphorical, ineffective but profitable “wars” on drugs, terror, and so on.

For a while, some commentators held that the rise of “finance-led growth” following the crisis of “wage-led growth” linked to Fordism would facilitate economic democracy. This would not involve workers’ control but enable citizen-clients to become sovereign-consumers. The Fordist wage relation was a key driver of the virtuous circle of mass production and mass consumption in relatively closed national economies. In contrast, the financialization of capitalist relations, including the wage relation, was supposed to enable workers to boost their incomes and wealth through home ownership, shares, funded private pensions, etc. (Aglietta and Rebérioux 2005; Boyer 2000). Some even believed that this “private Keynesianism” could replace the Keynesian national welfare state – but are less confident about this after the North Atlantic Financial Crisis (Crouch 2009). Well before this, however, other theorists already spoke of “finance-dominated accumulation” rather than “finance-led growth” in order to separate the empirical trend towards the autonomization of finance from the issue of whether it leads to growth, greater volatility, or stagnation (Stockhammer 2011, p. 3; see also van Treeck 2008).

The disembedding of capital from the frictions of national power containers and national politics means that the law of value tends increasingly to operate globally by commensurating local conditions at the same time as it promotes the treadmill search for superprofits. Interest-bearing capital gains strongly from world market integration because it controls the most liquid, abstract, and generalized resource and because it has become the most integrated fraction of capital. Moreover, as the world market gets more integrated and the space of flows, including finance, grows more important relative to territorially-delimited economic activities, challenges mount to the territorial and temporal sovereignty of states, whether or not these are democratic in substance or form.

As financialization expands and penetrates deeper into the social and natural world, it transforms the micro-, meso- and macro-dynamics of capitalist economies. Three basic manners of penetration can be distinguished. First, financialisation alters the calculations and behaviour of non-financial firms through the rise of shareholder value as a coercive discourse, technology of governance, and vector of competition. One aspect thereof is the growing importance for *non-financial* firms of financial activities that are not directly tied to their main profit-producing pursuits: for example, treasury functions, financial intermediation, using retained profits for share buybacks and/or acquisition or expansion of financial subsidiaries, etc. (Krippner 2005; Nölke 2009; Lapavitsas 2013). Second, it boosts the size and influence of the financial sector. Fee-producing and risk-taking activities increase relative to banking capital’s more traditional roles in intermediation and risk management – Deutsche Bank employees can write a book about it. Furthermore, securitization, leverage and shadow banking with corresponding liquidity risks and weak prudential controls expand, and so does the significance of new forms of financial capital like hedge funds, private equity, vulture capital and sovereign wealth funds. Third, as successive crises from the mid-1970s show, financialization makes the economy more prone to recession and, in severe cases, more liable to the downward spiral of debt-deflation-default dynamics (Dore 2008; Duménil and Lévy 2011; Fine 2010; Lapavitsas 2013; Rasmus 2010). Indeed, as more scandals emerge in the financial sector, it is becoming clear that these superprofits derive in part from predatory and, indeed, criminal activities that were facilitated by successive measures of deregulation enacted thanks to the financing of political parties and political corruption (Smith 2010; Will et al. 2013; Black 2015).

The best possible political shell for such a regime, more noted for its absence than presence, would be an ordoliberal framework, as envisaged in the original Social Market Economy paradigm. This would provide a formally adequate institutional and spatio-temporal fix, including the embedding of neoliberalism internationally in a new constitutionalism with credible commitments to corporate social responsibility. However, the neoliberal bias towards deregulation, which widened the space for financialization, was more often linked to an institutional fix that relied, and still relies, on “deals with political authorities”, predatory capitalism, and reckless speculation. These trends undermine the relation between free markets and democracy and have also fuelled the North Atlantic financial crisis.

Because continued financialized expansion depends heavily on the pseudo-validation of highly leveraged speculative and Ponzi debt, this regime contains its own inherent crisis-generating mechanism rooted in the systemic conflict between interest-bearing and profit-producing capital (cf. Hudson 2015). Wolfram Elsner (2012) explains this as follows: Financial capital in this regime has a target rate of return that is several times greater than the historic norm for profit-producing capital and, worse still, striving to achieve this target, engages in massive leveraging of fictitious credit and capital. In aggregate, the eventual validation of this massively leveraged capital would demand a total volume of surplus-value that far exceeds the productive and exploitative capacity of existing profit-producing capital.

Attempts to square this circle depend on three strategies that are individually and collectively unsustainable. One is to create and manage bubbles, the main redistribution mechanism in finance-dominated accumulation, and then bail out – or, rather: get bailed out – at the right moment (Elsner 2012, pp. 146 f.). Rather than allowing market forces to discipline financial institutions through losses and bankruptcy, states have socialized losses, translating private debt into public and/or sovereign debt. This is impossible without the complicity of central banks and government in the finance-dominated economies and those subject to their contagion effects. They have also taken direct responsibility for managing contagion effects in the always-already monetized “real economy”, albeit in the pro-cyclical, counterproductive form of private and public austerity. These measures have been pursued at national level by “natural governing parties” from the centre-left and centre-right as well as by more “technocratic” – that is, bank-friendly and non-accountable – regimes at the international, European, and some national levels. Indeed, the Federal Reserve, the Bank of England, and the European Troika, consisting of ECB, EU and IMF, asymmetrically defend too big to fail institutions, protect tax-avoiding, tax-evading companies and wealthy elites, and impose the costs of crisis on the general population (Johnson 2009; Hudson 2011; Elsner 2012). This has involved virtual *coups d’état* in Greece and Italy and the more general fuelling of “deficit hysteria” to justify yet more neoliberal policy measures in other indebted economies. Part of the ideological campaign in this regard is the spurious conflation of necessary but “boring” banking with financial speculation and risk-taking in the search for higher profits (LiPuma and Lee 2004; Haldane 2012; Elsner 2012).

A second strategy is to invoke a system-threatening “financial emergency” that justifies efforts to reduce individual and social wages, impose internal devaluation, and privatize public services and assets to pay off the public debt incurred in massive bailouts. States at different sites and scales have key roles here too. In the era of finance-dominated accumulation, taming the wilder manifestations also means to “intervene periodically to underwrite the solvency of banks, to provide extraordinary liquidity and to guarantee the deposits of the public with banks” (Lapavitsas 2013, pp. 27 f.).

The third strategy involves primitive accumulation through dispossession. Relevant techniques include land-grabbing, capitalizing nature and its services, enclosing the intellectual commons, privatizing accumulated public wealth, colonizing the residual public sector, and so on. This is also impossible without state involvement. In short, the most rarefied and leveraged forms of fictitious credit and capital are now primarily, and systemically rather than merely contingently, problem-makers – and the rest of the economy, society and nature are the problem-takers (Dörre 2012).

## The Challenges of Political Restoration and Social Emancipation

The form of the current democratic crisis differs from that envisaged in Marx’s comments on the fundamental contradiction in the democratic constitution. What is being restored politically as well as socially are not the privileges of some national Ancien Régime. Instead, we are seeing the political consolidation of a “new transnational power bloc” organized around interest-bearing capital. This bloc’s survival depends heavily on Weber’s three forms of political capitalism – that is, predatory profit from political activities, politically guaranteed monopolies and reliance on extraordinary political favors. The longer this bloc survives, the more it damages the nature, the “real economy”, and human flourishing. The post-democratic, authoritarian state of political emergency that is now being constructed will continue as the “political shell” for a predatory, finance-dominated accumulation regime even if, and when, the financial crisis is resolved.

Contrary to widespread assumptions of a fundamental contradiction between market forces and the state, this “new normal” regime both strengthens and weakens state power. On the one hand, the resort to bailouts and quantitative easing relies on the state’s role as lender of last resort. Along with its monopoly of taxation, this *does *indicate the limited powers of the state to tame the effects of crises and, thanks to the influence of financial interests, to introduce reforms that would present its resurgence. On the other hand, the unprecedented fisco-financial measures have failed to resolve the underlying contradictions and crisis-tendencies of finance-dominated accumulation: there is a palpable crisis of crisis-management in many states and international agencies. One sign of this is the growing split between the exit strategies proposed by profit-producing capital and the policies favoured by those identified with the more fantastical, irrational forms of interest-bearing capital and their allies.

In total, the neoliberal project has produced crises on three levels that are now shaking the pillars of Western societies: first, and most visible, a representational crisis as the electorate grows more detached from stable alignments with natural governing parties. Second, and more latent, a legitimacy crisis in several countries, following from the failure to deliver sustainable finance-led growth and the costs associated with crisis-management. Third, but not least, a crisis of intellectual and moral leadership associated with outright deception, official secrecy, populist rhetoric, and media spin. An initial response to the crisis in attempts to promote “more market, less state” was, and is, growing resort to flanking and supporting measures to keep the neoliberal show on the road. This was reflected in, inter alia, the discourse and policies of the “Third Way” or “Neue Mitte”, which sought to maintain the course of neoliberalization in new circumstances.

Furthermore, as neoliberal, “Third Way”, or more recent austerity policies begin to bite, there is growing, if still fragmented, resistance and growing anger about economic and political linkages among interest-bearing capital, politicians, and state managers. The best-known expressions of this resentment were for a while the Occupy Movement with its slogan of the 99% the 1% and the Astroturf “Tea Party” movement in the U.S. The latter is now mainstreamed in the Republican Party under Trump and has been absorbed into Trump’s “base”. But there are many other grass-roots manifestations in, for example, Greece, Spain, and Italy and many commentators have related the “Arab Spring” to the impact of neoliberal policies and financialization in the Middle East and North Africa. But these movements mostly operate at a distance from the state, changing the calculations of economic and political elites, but lacking access to the real levers of power in the circuits of capital, the inner sanctums of national and supranational state authorities, and the international agencies that exercise decisive private authority in the world market. Indeed, as one aspect of authoritarian statism, popular movements far removed from any conventional definition of terrorism are now targeted as if they were terrorist organizations. Anti-war, Occupy, environmental and animal rights movements are affected, while journalists and individual dissenters confront suspicion, surveillance, and intimidation (Boukalas 2014). It remains to see whether the many fragmented forms of resistance can be linked up horizontally, vertically, and transversally to provide an effective challenge to this new bloc, its finance-dominated accumulation regime and its “new normal” state form by exploiting their fragilities. This will require connecting economic and political power in ways that are “proscribed” by the democratic rules of the game but are realized continually in non-democratic ways by the new transnational financial bloc.

Overall, this reconfiguration of the state and governance in response to recent economic and political crises goes beyond the trends discussed in the 1920s and 1930s or the 1970s and 1980s. Nonetheless, the trends towards authoritarian statism have made it easier for capital to address the crisis of finance-dominated accumulation. It provides important precedents for the *de facto* declaration of a state of economic emergency that has justified both the use of exceptional powers to rescue insolvent financial institutions, rather than to nationalize them or allow normal bankruptcy procedures to be implemented, and the parallel declaration of a state of political emergency that justifies increased surveillance, pre-emptive policing, and paramilitary suppression of dissent. Even if there is a return to “financial business as usual” and interest-bearing capital has been fully restored *socially*, political restoration of democratic rule will not be volunteered by the financial oligarchy. It must be seized back from below.

## Towards an Enduring Austerity State

In spite of sharing a common gestalt, austerity policies differ in their concrete shape. That’s not only due to “varieties of capitalism”, reflecting their specific economic profiles and imaginaries: they are also shaped by interdependencies that result from interstate relations, including forms of regional and global governance, foreign trade plus other features of world market integration, and the prevailing logic of the world market. This highlights the need to examine austerity in terms of the relations between the economic and political fields, including their basic forms and institutional architecture, and their mediation through the changing balance of forces.

The well-known policy-politics-polity triplet suggests that austerity can be studied in three ways. First, there are *conjunctural austerity policies* that are introduced in the first instance as temporary measures in response to short-term or immediate problems. As the conjuncture becomes favourable again, these policies are suspended or reversed. Second, there is the *enduring politics of austerity*, often called “permanent austerity” in the relevant literature, that is promoted in response to a “chronic” crisis, real or manufactured, in the fisco-financial domain and/or in the economy more generally. This enduring politics of austerity, as noted above, is intended to bring about a more lasting reorganization of the balance of forces in favour of capital rather than to make policy adjustments to safeguard existing economic and political arrangements. Third, there is the *austerity polity*. The latter results from a continuing fundamental institutional reorganization of the relations between the economic and political in capitalist formations. It can be a possibly unintended cumulative result of the enduring politics of austerity, especially where this aggravates the underlying causes of fisco-financial crisis. But it can also result from a deliberate strategy to subordinate the polity more directly and durably to the “imperatives” of the world market as these are construed in neoliberal discourse with its one-sided emphasis on the logic of exchange-value. And, given the political, ideological, hegemonic, and organic crises that have developed in the context of the financial, economic, and fisco-financial crises, the austerity polity can also be an authoritarian response to growing popular unrest – including right-wing extremism – about the technocratic and plutocratic nature of crisis responses (cf. Jessop 2015a).

Whereas conjunctural policies are found in the pattern of neoliberal policy adjustment and associated with targeted cuts in specific areas, an enduring politics of austerity is characteristic of neoliberal regime shifts and assumes the form of general fisco-financial restraint, putting downward pressure on most areas of expenditure, especially discretionary ones (Pierson 2002; Ferrera 2008; Seymour 2014). This pattern can occur in normal forms of politics, in states of economic emergency or, even, in lasting states of exception. It can be triggered by an obvious crisis, one that is deliberately exaggerated, or one “manufactured” for political purposes. Indeed, in neoliberal regimes, whatever the state of the economy, it seems that it is always the right time to reduce public expenditure – except for corporate welfare – through an appropriately crafted and crafty politics of austerity. This involves far more than quantitative cuts in spending because it is also intended to have qualitative, transformative effects. It is pursued as a means to consolidate and extend the power of capital, especially interest-bearing capital, and to subsume ever wider areas of social life under the logic of differential accumulation. It becomes a major vector of the colonization, commodification, and, eventually, financialization of everyday life – processes subject to friction, resistance, and crisis-tendencies.

Over the past decade, similar ideas have been developed by other critical commentators, from the right as well as the centre and left, especially in the context of the recent and continuing financial crisis and its broader economic repercussions. For example, Greg Albo and Carlo Fanelli (2014) refer to a new phase of bipartisan or pluripartisan “disciplinary democracy” as the political form of “permanent austerity” (Rasmus 2010; Stützle 2013). Ian Bruff refers to neoliberal authoritarian constitutionalism (Bruff 2013), Ingar Solty (2013) identifies an authoritarian crisis constitutionalism oriented to the economic governance of competitive austerity, and Lukas Oberndorfer describes the development of authoritarian competitive statism (2015). From a social democratic perspective, Wolfgang Streeck refers to a move from the welfare state to the consolidation state (2014). And a (former) Fabian Socialist, Colin Crouch, describes the transition to post-democracy (2008). On the libertarian right, there is condemnation of the strong and repressive state that emerges from allegedly unconstitutional intervention to shore up finance capital and police dissent (e.g. Stockman 2013). Such claims prompt the question whether these are short-term aberrations, conjunctural states of emergency, or precursors of a “new normal”.

Seymour (2014) argues for the latter: in his view, austerity involves something much broader and more complex than spending cuts – thanks to its role in restructuring, recalibrating, and reorienting state expenditure. Indeed, for him, austerity is *the* dominant political articulation of the global economic crisis in Europe and North America. This strategy, he argues, has seven aspects: first, to rebalance the economy from wage-led to finance-led growth; second, to redistribute income from wage-earners to capital; third, to promote ‘precarity’ in all areas of life as a disciplinary mechanism and means to reinforce the financialization of everyday life; fourth, to recompose social classes, with growing inequality in income and wealth and greater stratification within classes; fifth, to facilitate the penetration of the state by corporations; sixth, to accelerate the turn from a Keynesian welfare state based on shared citizenship rights to a workfare regime that relies on coercion, casual sadism, and, especially in the US, penality; and, seventh, to promote the values of hierarchy and competitiveness (Seymour 2014, pp. 2 ff.).

In many respects, these aspects were already inscribed in the politics of neoliberal regime shifts. For Seymour, though, they were reinforced after the 2007–2009 financial and economic crisis. This can be explained in part by the fact that the painful measures already taken to consolidate budgets in the 1990s and early 2000s were wiped out by the impact of the North Atlantic Financial Crisis and the Eurozone crisis as governments took on more debt to bail out banks and/or create stimulus packages (Rasmus 2010; Hudson 2012).

This ramping up of the politics of austerity occurred in part because the response of financial capital to this crisis intensified the state’s fisco-financial crisis. Measures were taken to rescue interest-bearing capital from the effects of its Ponzi dynamic and from the inherently unsustainable drive for financial profits (see above; Demirović & Sablowski 2013). This created a debt-default-deflation dynamic that has worsened public finances as well as the private sector (Rasmus 2010). In addition, as Seymour, among others, notes, the politics of permanent austerity is not just a response to economic crisis but also to political and ideological crises and, indeed, an organic crisis of the capitalist social order (Seymour 2014, p. 4; Gramsci 1971; Bruff 2013). This is used to justify a state of economic emergency that is presented initially as a “temporary” response to immediate or chronic problems, but then acquires more permanent form through cumulative and mutually reinforcing institutional change, routinization of exceptional measures, and habituation.

The politics of austerity can, therefore, be interpreted as a long-term strategic offensive to reorganize the institutional matrix and balance of forces in favour of capital. It aims to rearticulate relations between both the social power of money as capital, respectively of capital as property on the one hand and the political power of the state on the other. Inter alia, this involves a politics aimed at *disorganizing* subaltern classes and *reorganizing* the capitalist power bloc around interest-bearing capital in neoliberal regimes and export-based profit-producing capital in economies where neoliberal policy adjustments prevailed. In the Eurozone, for example, the central goal of authoritarian crisis constitutionalism is to deepen EU integration on neoliberal terms and govern through competitive austerity. Its aims include socializing bank losses, exploiting the sovereign debt crisis to restructure welfare states and labour markets – including further measures to weaken trade union bargaining power – and to impose shock therapy in the periphery. In both finance-dominated and export-oriented regimes, the overall approach can switch between offensive and defensive tactics. An example of the latter is again the “Third Way”, with its flanking and supporting mechanisms to maintain the overall momentum of neoliberal transformation. The successful pursuit of this strategy, which cannot be taken for granted, leads to an *austerity state* embedded in a political system that institutionalizes a permanent politics of austerity.

As shown above, much of this was already revealed by Poulantzas when the current era of capitalist crises was only looming on the horizon. Writing in the mid-1970s, though, he largely neglected the development of authoritarian statist tendencies at the transnational level. Developments here involve scale-jumping for capital coordinated through parallel power networks and oriented to securing the conditions for a “new constitutionalism” (Gill 1995) that provides super-protection for capital as neoliberalism is rolled out globally and limits the territorial and temporal sovereignty of national states. The secret negotiations between national and EU administrations, the representatives of capital, and the Post-Washington Consensus international economic institutions around the Trans-Pacific Partnership (TTP) and the Transatlantic Trade and Investment Partnership (TTIP) are an extreme example of this trend. They aim to re-scale quasi-constitutional protections for capitalist enterprises and their activities to the international level – thereby removing them from the more contentious field of national politics –, to allocate adjudication over disputes, including with states, to private tribunals, experts, lawyers, and other ostensibly non-political forums and/or figures, and, surprisingly (or not) in allegedly democratic regimes, to limit the power of elected governments to introduce legislation or administrative rules that would harm the anticipated profits of transnational enterprises.

The growing popular hostility to TPP and TTIP as details leak into the public domain is one illustration of the limits of the power of the transnational deep state. Another is the growing concern among economic and political elites about the backlash from growing inequalities of wealth and income and the obvious bias in favour of financial capital in managing the North Atlantic financial crisis and the Eurozone crisis. Thus, the rise of “authoritarian statism” involves a paradox. While it clearly strengthens state power at the expense of liberal representative democracy, it also weakens its capacities to secure bourgeois hegemony (Poulantzas 1978, pp. 241, 263 ff.; Bruff 2013).

## Conclusions

Drawing on the work of Marx, Gramsci, and especially Poulantzas, I have argued that the relation of formal adequacy between capitalism and democracy is limited to what Weber calls “rational capitalism” and depends on the temporary, provisional resolution of the “comprehensive contradiction” at the heart of the democratic constitution. The approach developed above emphasizes the need for political analysis to consider state forms, political regimes, political discourses, and the changing balance of political forces as well as basic economic relations, economic crises, and the economic conjuncture. This supports three claims about the historically contingent nature of the relation between capitalism and democracy. First, finance-dominated accumulation is associated with new forms of political capitalism that facilitated its rise, enable its expansion, and intervene to resolve its crises in the interests of financial capital. Second, the institutional separation of the economic and political spheres that characterized liberal democracy is in decline and leads to the intensification of “exceptional” elements of the state as dominant fractions of capital and state managers seek to address the crisis-tendencies of finance-dominated accumulation and resistance to neoliberalization. Third, these trends are connected with the neoliberal politics of austerity and the rise of the austerity state. Overall, this suggests that the subaltern classes have lost the battle to prevent the political restoration of capitalist domination for the time being.

Whether they can reverse this and engage in a war for social emancipation remains to be seen. In the meantime, the preceding arguments indicate three areas for further theoretical reflection and/or political action. The first concerns the limits of finance-dominated accumulation as opposed to financialization more generally. This has several aspects: the limits to the continued expansion of fictitious credit, fictitious capital, and fictitious profits at the expense of profit-producing capital^5^ and the growing indebtedness of workers and households as real wages stagnate; the diminishing room for manoeuvre of financial authorities when the next financial crisis erupts, especially as the concentration of interest-bearing capital has intensified after the North Atlantic financial crisis; the declining competitiveness of finance-dominated economies relative to economies where profit-producing capital remains dominant – notably in some Rhenish capitalist economies and China, with its 2025 technology plan; and the rise of right-wing and left-wing populism among social categories that are real or perceived losers from finance-dominated accumulation and neoliberalization more generally.

The second area for further theoretical work concerns the limits to the permanent austerity state as the current form of authoritarian statism. There is a risk here that the response to the next major financial crisis will be increased austerity unless it is not managed like the last major crisis to rescue interest-bearing capital at the expense of other fractions and the subaltern classes. There are crucial current and future limits to further intensifying austerity. For, given market failures, the bias of neoliberalization to profit-maximization to the neglect of substantive negative externalities across different aspects of the capital relation and wider social formation (on which, see Jessop 2012), and growing disaffection with austerity and its social consequences, further austerity – especially if intended to socialize the losses of capital – will reinforce the backlash against the austerity state. The political challenge regarding the failures of finance-dominated accumulation and the limits of further austerity is how to counteract the rise of right-wing populism and mobilize resistance towards projects of social emancipation oriented to human flourishing and environmental justice.

The third area is more political but should rest on theoretically-informed conjunctural analysis. It concerns the forms, stakes, sites, and social bases of resistance to the trends explored above. Here we should first note that resistance is not reactive but is an important driving mechanism behind the emergence and changing character of all three trends. This applies especially to neoliberalization, which emerged in part in response to conflictual crises of Atlantic Fordism, state socialism, and import-substitution industrialization and which changes shape as it “fails forward” (Peck 2010, p. 6) and encounters new forms of resistance (Featherstone 2015). Finance-dominated accumulation in turn could mobilize resistance from larger profit-producing and commercial capitals, small and medium enterprises, and subaltern groups exploited through usury and facing mounting indebtedness and stagnant wages. And the permanent austerity state, with its “exceptional” elements, is a response to the intertwined constraints of the neoliberal privatization of profits and the socialization of losses – especially for predatory financial capital –, neglect of the material and institutional conditions of existence of profitable accumulation at the expense of short-term profit maximization, and growing but fragmented resistance to austerity and exclusion. The respective crises and struggles in each case are quite heterogeneous, with different symptoms and results for different social forces and social categories in different contexts, and do not and cannot generate a homogeneous response. The complex symptomatology of crises creates multiple challenges over how to define the symptoms, construe their underlying causes, and translate these diagnoses into remedial or palliative actions and, worse, if they are misdiagnosed, actions that aggravate or intensify underlying causes and symptoms (on the challenges of symptomatology, see Jessop 2015b).

So far, the right in various guises has managed to interpret the multiple, heterogeneous, and interconnected crises of neoliberalization, finance-dominated accumulation, and enduring austerity in terms favourable to capital and/or right-wing populism that disorganizes the left. As yet, then, the political restoration of capitalist domination in neoliberal regimes has not created the conditions for social mobilization oriented to the social emancipation of subaltern classes and groups. New ecological, economic, political and social imaginaries that can construe the intensifying crises are crucial here. Whether they can be developed through connecting grass-roots resistance across different territories, places, scales, and networks and building on local experiences, or through broader initiatives aimed at building political, intellectual, and moral leadership through more encompassing political parties and social movements remains the key strategic dilemma for organizing resistance. The challenge is how best to develop new forms of social organization – or an ecology of organizations – that can address both horns of this dilemma. This is not the topic for another scientific paper but for actual and potential social movements.

## Footnotes

1 Marx analysed formally rational capitalism in *Das Kapital*. It seems that he wanted to show that a mode of production characterized by “freedom, equality, property and Bentham” *in the labour market* (Marx 1972, p. 189) was fully compatible with economic exploitation, political domination, contradictions, and crisis-tendencies in the overall mode of production. Elsewhere, Marx did analyse forms of political capitalism, including mercantilism, colonialism, primitive accumulation, Louis Bonaparte’s political regime, and so on. And he also showed explicitly or implicitly how these were formally and substantively incompatible with liberal democracy (on Bonapartism, see Marx 1980).

2 Following Swedberg 1998, I consider these as forms of political capitalism (pp. 48 ff., 172).

3 Related literature points to the possibility of more enduring forms of dictatorship. This is reflected in two recent lines of state-theoretical analysis: a soft thesis about the continuing decline of liberal democracy and a strong thesis about the irresistible rise of authoritarian statism (compare, for example, Crouch 2008 and Streeck 2014 with Poulantzas 1978, Bruff 2013, and Oberndorfer 2015; for further discussion, see below). Whereas the former thesis tends to focus on symptoms at the level of the political scene, the latter tends to ground its analysis in more fundamental shifts in contemporary capitalism and challenges to national security – given that the national security apparatus links different apparatuses within the state, operates across its formal boundaries with society, and is itself linked through parallel power networks to important forces beyond the state. For a comprehensive analysis of the current global re-emergence of Bonapartist politics see Beck and Stützle 2018; for the British case in particular Jessop 2018 therein.

4 The current Trump Administration illustrates some of these tendencies. Yet, its actions to date seem inconsistent with others. The state administration remains the central site at which conflicts in the power bloc are expressed, and there is a notable tightening of the ties to corporate America within and beyond Trump’s so-called “billionaires’ Cabinet”. Politics is also increasingly focused in and on the President’s staff office. However, the latter is dysfunctional, staff turnover is high by historical standards, policy-making is often chaotic, prone to grand but ineffective and reversible gestures, including publicity-generating executive decisions that fall foul of remaining constitutional checks and balances, and lacks a sense of strategic direction. There is little strategic effort to secure support in Congress for Trump’s legislative proposals and/or reconcile the interests of conflicting capital fractions nationally and internationally. Moreover, rather than re-balancing conflicting social forces to secure social cohesion, Trump employs faux populism, alt-right social media, and dog whistle politics to maintain the support of his “base” at the same time as encouraging voter suppression against potential Democrat electors. This said, while there is no coherent *positive* strategy to reorganize the power bloc, a series of deliberately destructive steps is advancing the specific interests of different capital fractions, including his own family businesses. These steps include budget and staff cuts plus non-enforcement or deregulation in many regulatory agencies, the packing and “weaponizing” of courts, and removing the half-hearted restrictions and capital requirements on financial capital introduced by the Wall Street Reform and Consumer Protection (or Dodd-Frank) Act after the 2007–2008 financial crisis. Along with tax cuts and other largesse for corporations and ultra-high net worth families, these measures will entrench corporate power and privilege. At the same time there is further sabotaging of the welfare state in areas such as education, health, housing, social security, and other safety nets and attacks on labour rights. These policies reinforce austerity and require further entrenchment of the surveillance state, including, under Trump, intimidation of the mainstream mass media as purveyors of “fake news” and “enemies of the people”. Many measures coincide with the neo-conservative and neoliberal agenda of billionaires such as the Koch brothers and as relayed through multiple lobbying groups. Key beneficiaries in the short- to medium-term are the fossil fuel industries, the military-industrial complex, and financial capital.

5 This can be seen in, inter alia, the use of profits and tax breaks to buy back shares rather than invest in research and innovation to maintain competitiveness; this is a form of self-cannibalization of profit-producing capital.
